# Risk Factors for Early Shoulder Stiffness After Arthroscopic Rotator Cuff Repair

**DOI:** 10.7759/cureus.93335

**Published:** 2025-09-27

**Authors:** João Felipe M Filho, Rodrigo A Beraldo, Mauro E Gracitelli, Jorge Assunção, Nuno Sevivas, Eduardo A Malavolta

**Affiliations:** 1 Orthopedics and Traumatology, Universidade Federal do Rio Grande do Norte, Natal, BRA; 2 Orthopedics and Traumatology, Instituto Jundiaiense de Ortopedia e Traumatologia, Jundiai, BRA; 3 Orthopedics and Traumatology, Hospital das Clínicas HCFMUSP, Faculty of Medicine, University of São Paulo, São paulo, BRA; 4 Orthopedics and Traumatology, Hospital das Clínicas, Faculty of Medicine, University of São Paulo, São Paulo, BRA; 5 Orthopedics and Traumatology, Centro Hospitalar do Médio Ave, Braga, PRT; 6 Orthopedics and Traumatology, Hospital das Clínicas HCFMUSP, Faculdade de Medicina, Universidade de São Paulo, São Paulo, BRA

**Keywords:** arthroscopic rotator cuff repair, arthroscopy, postoperative complications, risk factors, shoulder stiffness

## Abstract

Introduction: Arthroscopic rotator cuff repair (ARCR) is the gold standard for treating symptomatic rotator cuff tears. Despite its benefits, early postoperative shoulder stiffness (EPS (shoulder)) remains a concern, with reported incidence varying widely. Identifying risk factors is crucial for optimizing outcomes. This study aimed to test whether preoperative clinical comorbidities and MRI-based structural tear features are associated with EPS after ARCR, where EPS was the primary outcome (present/absent) defined as persistent passive range of motion (ROM) limitation >90 days postoperatively.

Methods: This retrospective cohort study analyzed 381 patients who underwent primary ARCR at a tertiary referral center between December 2017 and February 2022. Data were collected retrospectively through electronic medical records and managed in a secure institutional database. Patients with preoperative stiffness or requiring additional procedures such as biceps tenodesis, capsular release, or distal clavicle resection were excluded. Clinical variables, including age, sex, diabetes, arterial systemic hypertension, dyslipidemia, and hypothyroidism, were assessed. Rotator cuff tears characteristics were analyzed through preoperative MRI and categorized using the Snyder, Cofield, Patte, and Goutallier (Fuchs modified) classifications.

Results: EPS was observed in 6.3% (24/381) of patients. The mean age of patients with EPS was 57.4 ± 11.1 years, compared to 59.9 ± 9.5 years in the non-stiffness group (p = 0.056). Women represented 75% of the EPS group versus 60.8% in the non-EPS group (p = 0.242). No significant associations were found between EPS and diabetes (p = 0.592), hypertension (p = 0.271), dyslipidemia (p = 0.220), or hypothyroidism (p = 0.999). Regarding anatomical characteristics, 87.5% of EPS cases involved full-thickness tears, compared to 80.1% in the non-EPS group (p = 0.592). Tear size, tendon retraction, and fatty degeneration showed no significant association with EPS.

Conclusion: No clinical or structural variables were significantly associated with EPS after ARCR. Standardized criteria are needed to identify high-risk patients and refine preventive strategies.

## Introduction

Rotator cuff repair, mainly through arthroscopic techniques, had become the gold standard for treating rotator cuff tears refractory to conservative management [1]. Over the past decades, advancements in surgical techniques and increased access to specialized services have significantly increased the number of procedures performed worldwide [2-4]. This intervention modifies the natural history of the disease by preventing lesion progression and improving patients' quality of life [5,6].

Despite its benefits, complications such as early postoperative stiffness (EPS) remain a significant concern. This condition, characterized by a reduction in shoulder range of motion (ROM) during the first months after surgery and delaying the patient’s recovery, has an incidence ranging from 2.3% to 31%, depending on the diagnostic criteria and assessment time points [7-9]. It can compromise shoulder function, negatively impact patients' daily activities, and, in some cases, require additional interventions such as manipulation under anesthesia or capsular release [8,10].

Patient characteristics, such as female sex, age under 50, diabetes mellitus, and hypothyroidism, along with surgical technique and postoperative management, contribute to the development of stiffness [6,9,11]. Although studies have investigated prognostic factors for clinical and structural outcomes after rotator cuff repair, the literature remains inconclusive regarding which factors most determine early stiffness [12,13]. This uncertainty hinders the identification of at-risk patients and the implementation of effective preventive strategies [9,11].

This study aimed to evaluate the clinical and structural characteristics of developing EPS in patients undergoing arthroscopic rotator cuff repair (ARCR).

Our primary outcome was EPS (present/absent) defined as persistent passive ROM limitation beyond 90 days after ARCR; predictors included age, sex, diabetes, systemic arterial hypertension, dyslipidemia, hypothyroidism, and MRI classifications (Snyder, Cofield, Patte, Goutallier).

## Materials and methods

Study design and setting

A retrospective cohort study based on prospectively collected data from patients who underwent ARCR at a high-volume referral center between December 2017 and February 2022. The study was approved by the Institutional Research Ethics Committee (Universidade Federal do Rio Grande do Norte, protocol 74025823.9.0000.5292).

Inclusion and exclusion criteria

We included patients over 18 years of age diagnosed with partial or full-thickness tears of the posterosuperior rotator cuff tendons who underwent primary arthroscopic repair and with a minimum follow-up period of 24 months. We did not include patients with preoperative stiffness.

Exclusion criteria comprised irreparable tears identified intraoperatively or the need for concomitant procedures such as biceps tenodesis, capsular release, or distal clavicle resection. Patients with a clinical follow-up of less than six months were excluded.

Definition and assessment of postoperative stiffness

EPS was defined as the persistent limitation of passive ROM, measured with a goniometer and assessed by a single examiner-an orthopedic surgeon with over 20 years of experience.

Diagnostic criteria included passive external rotation of less than 10° with the arm at the side or less than 30° in 90° abduction or passive forward flexion of less than 100°, persisting for more than 90 days after surgery, as previously defined by Brislin et al. [14].

Studied variables and data collection

Clinical factors such as hypothyroidism, diabetes mellitus, systemic arterial hypertension, and dyslipidemia were assessed. These conditions were confirmed through a review of medical history and assessment of the use of specific medications.

Structural factors were obtained from preoperative MRI scans, which were evaluated by a team of radiologists with over 10 years of experience in musculoskeletal imaging using standardized classification systems.

The Snyder classification was used to assess the extent of the tear, categorizing lesions as partial (types A and B) or complete (type C) [15]. In this study, patients were grouped into these two subgroups to explore potential differences in the incidence of stiffness between partial and complete tears based on their association with tear patterns reported in the literature.

The Cofield classification was used to categorize tear size into four groups: small (<1 cm), medium (1-3 cm), large (3-5 cm), and massive (>5 cm) [16]. For statistical analysis, patients were grouped into small/medium tears and large/massive tears.

The Patte classification was used to determine the degree of tendon retraction [17]. This system categorizes cases into three grades: Grade I, where retraction is limited to the greater tuberosity; Grade II, where retraction reaches the humeral head; and Grade III, where retraction extends beyond the humeral head, typically at the level of the acromion. For statistical analysis, patients were grouped into mild retraction (Grades I and II) and severe retraction (Grade III) to investigate whether the severity of retraction influenced postoperative stiffness.

Finally, the Goutallier classification was used to assess fatty degeneration of the rotator cuff muscles, categorizing it into grades from 0 to 4 [18]. Grade 0 indicates the absence of degeneration, while grade 4 reflects severe degeneration. For analysis purposes, patients were grouped into two subgroups: no degeneration (grade 0) and mild to severe degeneration (grades 1 to 4), aiming to explore the association between fatty degeneration and the development of stiffness.

Data collection was performed retrospectively through electronic medical records, complemented by manual review when necessary.

Surgical protocol and rehabilitation

The same specialist, who has over 20 years of experience, performed all surgeries. Procedures were performed with patients in the lateral decubitus position under general anesthesia combined with an interscalene block. Systematic bursectomy and debridement of the greater tuberosity were performed. Rotator cuff repair was conducted using the double-row technique for full-thickness and partial articular tears and the single-row technique for partial bursal tears. The number of anchors and the potential need for additional lateral sutures were determined intraoperatively.

Interventions on the biceps tendon were indicated in partial tears, subluxations, or complete dislocations. Tenotomy was preferred for patients over 60, while tenodesis was performed in younger individuals. When required, biceps fixation was performed using one of the anterior anchors positioned superior to the rotator cuff.

Pain management followed a standardized analgesic regimen. During hospitalization, patients received intravenous medication consisting of dipyrone (2 g every six hours), a non-steroidal anti-inflammatory drug (NSAID), ketoprofen (100 mg every 12 hours), and an opioid analgesic, tramadol (100 mg every 8 hours). After discharge, analgesia was maintained orally with celecoxib (200 mg every 12 hours) and paracetamol (500 mg every six hours) continuously, with codeine (30 mg every six hours) available for severe pain relief if necessary. This regimen was maintained for seven days and adjusted individually based on each patient's clinical response.

The postoperative protocol varied according to tear size. Patients with small tears were immobilized with a sling for four weeks, while those with medium to large tears remained immobilized for six weeks. The shoulder was restricted during the first two weeks, with no active or passive movement. From the second week, passive exercises were introduced. After removing the sling at four or six weeks, assisted active exercises began, followed by free active movements. Muscle strengthening was initiated in the 12th week.

Statistical analysis

A convenience sampling strategy was used, including all eligible patients who underwent surgery during the study period.

Categorical variables were expressed as absolute (n) and relative (%) frequencies. The normality of numerical variables was assessed using the Kolmogorov-Smirnov test, indicating a normal distribution's absence. As a result, numerical data were presented as mean and SD.

The chi-square test or Fisher's exact test, as appropriate, was used to compare categorical variables between groups. Additionally, a multivariate logistic regression analysis was performed including variables with p < 0.20 in univariate analysis to adjust for potential confounders. Odds ratios (ORs) with 95% confidence intervals (CIs) were reported. 

The relative risk (RR) and 95% CIs were calculated to evaluate the association between clinical and structural variables and EPS. An RR greater than 1 indicated an increased risk, while an RR less than 1 suggested a potential protective effect. Statistical significance was considered when the 95% CI did not include 1.

We used the SPSS software version 20.0 for data analysis, adopting a significance level of 5%.

## Results

A total of 381 patients who underwent ARCR were analyzed. Of these, 147 (38.6%) were men and 234 (61.4%) were women, with a mean age of 59.7 ± 9.6 years. The overall incidence of EPS was 6.3% (24 patients).

The mean age in the group that developed EPS was 57.4 ± 11.1 years, while in the non-stiffness group it was 59.9 ± 9.5 years, with no statistically significant difference (p = 0.056). Although not significant, patients under 50 years old had a higher proportion of EPS (29.2%) compared to the non-stiffness group (14.6%). Regarding sex, women represented 75% of patients with EPS compared to 60.8% in the non-stiffness group (p = 0.242), indicating a trend without statistical confirmation (Table 1).

**Table 1:** Baseline demographic and clinical characteristics of patients with and without EPS after ARCR Comparisons performed using chi-square test or Fisher’s exact test, as appropriate. Statistical significance set at p<0.05. EPS: Early postoperative stiffness; ARCR: Arthroscopic rotator cuff repair

Characteristic	Stiff (n = 24)	Non-Stiff (n = 357)	p-value
Age: ≥50 years	17 (71.8%)	305 (85.4%)	0.104
Age: <50 years	7 (29.2%)	52 (14.6%)	-
Sex: Female	18 (75.0%)	217 (60.8%)	0.242
Sex: Male	6 (25.0%)	140 (39.2%)	-
Diabetes mellitus	3 (12.5%)	71 (19.9%)	0.592
Systemic hypertension	7 (29.2%)	153 (42.9%)	0.271
Dyslipidemia	3 (12.5%)	90 (25.2%)	0.220
Hypothyroidism	3 (12.5%)	47 (13.2%)	0.999

Regarding comorbidities, diabetes mellitus was present in 12.5% of patients with EPS versus 19.9% of those without (p = 0.592). Systemic arterial hypertension was observed in 29.2% of patients with EPS compared to 42.9% of the non-stiffness group (p = 0.271). Dyslipidemia was present in 12.5% of EPS patients versus 25.2% in non-stiffness patients (p = 0.220). Hypothyroidism was found in 12.5% of EPS patients versus 13.2% in those without EPS (p = 0.999). None of these associations reached statistical significance (Table 1).

Analysis of anatomical factors showed that 87.5% of patients with EPS had full-thickness tears compared to 80.1% in the non-stiffness group (p = 0.592). Tear size according to Cofield’s classification showed 16.7% of EPS patients had large or massive tears compared to 23.8% of the non-stiffness group (p = 0.618). Regarding tendon retraction, severe retraction (Patte III) was present in 8.3% of EPS patients and 8.7% of non-EPS patients (p = 0.999). Fatty degeneration (Goutallier 1-4) was observed in 62.5% of EPS patients versus 72% in non-EPS patients (p = 0.560) (Table 2).

**Table 2:** Preoperative MRI-based structural characteristics of rotator cuff tears in patients with and without EPS Comparisons performed using chi-square test or Fisher’s exact test, as appropriate. Statistical significance set at p<0.05. EPS: Early postoperative stiffness

Classification	Stiff (n = 24)	Non-Stiff (n = 357)	p-value
Snyder C (Full thickness)	21 (87.5%)	286 (80.1%)	0.592
Snyder A/B (Partial)	3 (12.5%)	71 (19.9%)	-
Cofield small/medium	20 (83.3%)	272 (76.2%)	0.618
Cofield large/massive	4 (16.7%)	85 (23.8%)	-
Patte I/II (Mild retraction)	22 (91.7%)	326 (91.3%)	0.999
Patte III (Severe)	2 (8.3%)	31 (8.7%)	-
Goutallier 0	9 (37.5%)	98 (28.0%)	0.560
Goutallier 1–4	15 (62.5%)	257 (72.0%)	-

**Figure 1:**
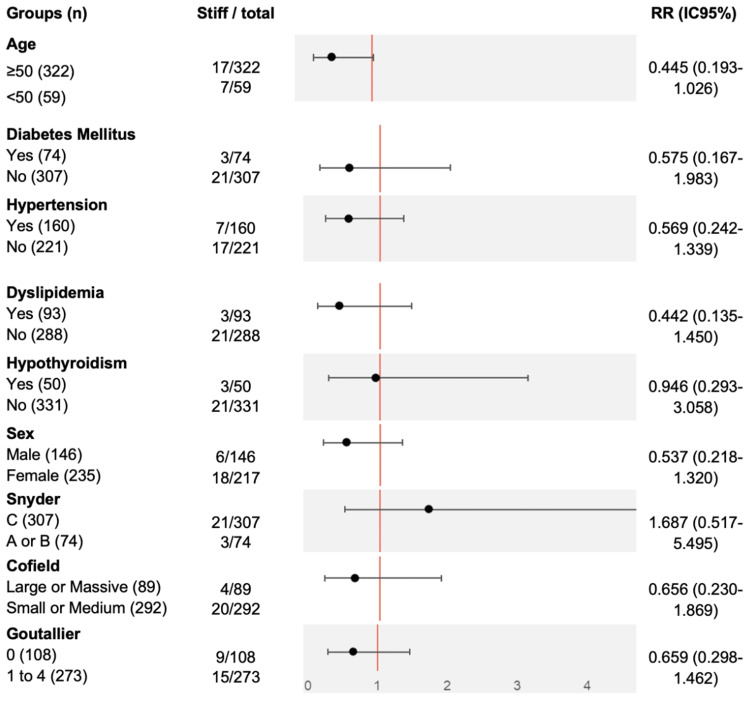
RR of clinical and structural factors associated with EPS (shoulder) Risk ratios and 95% CIs were calculated. Statistical significance set at p<0.05. RR: Relative risk; EPS: Early postoperative stiffness; CI: Confidence interval

Overall, none of the analyzed clinical or anatomical variables demonstrated a statistically significant association with EPS (Figure 1).

In multivariate analysis, none of the variables (sex, age <50 years, partial tears) remained significantly associated with EPS.

## Discussion

Our study did not identify statistically significant associations between the evaluated clinical or structural factors and the development of EPS after ARCR. Multivariate analysis also did not identify independent predictors of EPS, which strengthens the interpretation that no isolated factor had a significant impact in this cohort. The incidence of EPS was 6.3%, which is consistent with the findings of Baumann et al., who reported an average rate of 6.4% in a 2023 systematic review encompassing 9.373 patients [19]. Despite this, the literature shows a wide variability in stiffness rates, ranging from 2.3% to 31% [11,20]. This discrepancy is often attributed to the heterogeneity in the definitions of stiffness and the different postoperative assessment time points used across studies. Additionally, population and methodological differences highlight the complexity of this issue, reinforcing the need for standardized criteria to identify risk factors and enable comparisons between studies.

Our study did not identify a significant association between female sex and the risk of EPS, with a prevalence of 75% among patients with stiffness compared to 60.8% in the non-stiffness group (p = 0.242). These results differ from those of Blonna et al., who reported female sex as a significant risk factor for severe stiffness (RR = 7.6; p = 0.04) [20]. Similarly, Cucchi et al. observed a higher incidence of stiffness in women after the repair of partial tears (p = 0.0005) [21]. Conversely, studies by Tan et al., Salas et al., and Baumann et al. did not find a statistically significant association between sex and postoperative stiffness, which aligns with our findings [7,19,22].

Age was not a significant factor in our analysis (p = 0.104), consistent with findings from similar studies published in the literature [7,10]. In contrast, Chung et al. reported advanced age as a risk factor for stiffness, with statistical significance (p < 0.01 to p < 0.001) [8]. On the other hand, Guo et al. and Cucchi et al. observed a higher incidence of stiffness in younger patients (p < 0.0001 and p = 0.007, respectively) [11,21]. These discrepancies may be attributed to differences in diagnostic criteria and methodological approaches across studies, emphasizing the need for standardization in investigating the relationship between age and postoperative stiffness.

In our analysis, diabetes mellitus was not significantly associated with the development of postoperative stiffness (p = 0.592), which aligns with the findings of Tan et al., Salas et al., and Huberty et al. [7,10,22]. In contrast, Blonna et al. identified DM as a significant risk factor for moderate stiffness (RR = 5.7; p = 0.03) [20]. Additionally, Baumann et al. reported a relationship between DM and longer rehabilitation times (p < 0.01), though without directly impacting stiffness incidence [19]. These differences may reflect the inclusion of patients with subclinical or prediabetes in some studies, as observed by Blonna et al., who used detailed laboratory assessments to identify previously undiagnosed metabolic dysfunctions [20]. These findings highlight the need for future studies to evaluate the impact of different diabetes subtypes on the development of stiffness.

Hypothyroidism was not significantly associated with the development of postoperative stiffness in our analysis (p = 0.999), consistent with other authors who also found no statistical relevance for this variable [7,22]. In contrast, Blonna et al. identified hypothyroidism as a significant risk factor for severe stiffness (RR = 25; p < 0.001), suggesting that subclinical forms of this condition may contribute to this outcome in some patients [20]. These authors reported that 75% of patients diagnosed with hypothyroidism during the study developed postoperative stiffness, emphasizing the importance of screening for hormonal dysfunctions before surgery.

In our analysis, systemic arterial hypertension and dyslipidemia were not significantly associated with developing postoperative stiffness (p = 0.271 and p = 0.220, respectively). These findings are consistent with those of Tan et al. and Salas et al., who also found no statistical relevance for these conditions in their studies [7,22].

Diabetes mellitus, hypothyroidism, and dyslipidemia are well-established risk factors for adhesive capsulitis, an inflammatory condition that can lead to capsular fibrosis and shoulder stiffness, regardless of rotator cuff repair. This suggests a potential bias in studies on postoperative stiffness, as some patients may already have an underlying subclinical inflammatory process before surgery, even without a prior diagnosis of adhesive capsulitis. This hypothesis could contribute to the inconsistencies in the literature regarding the impact of these metabolic and hormonal disorders on postoperative stiffness. Additionally, the lack of standardized criteria to differentiate postoperative stiffness from adhesive capsulitis complicates the analysis of these risk factors and may affect the interpretation of results. Future studies investigating specific inflammatory markers and conducting more detailed clinical follow-ups are essential to clarify this relationship and identify patients at higher risk of developing stiffness.

The anatomical characteristics of the lesions, as assessed by the Cofield, Snyder, Patte, and Goutallier classifications, were not significantly associated with the development of postoperative stiffness in our analysis. These findings align with those of Salas et al. and Tan et al., who also found no statistical relevance for variables such as tear size or degree of tendon retraction [7,22]. In contrast, Cucchi et al. observed a higher incidence of stiffness in smaller and partial tears (p = 0.0083), while Chung et al. reported that larger tears were associated with greater stiffness at the final follow-up (p < 0.001) [8,21]. These discrepancies may be attributed to methodological differences, such as the use of varied classification cutoffs and the inclusion of different types of lesions across studies. The lack of association in our sample suggests that other factors, such as rehabilitative or inflammatory characteristics, may play a more decisive role in developing postoperative stiffness. Additionally, the lower number of patients with partial tears in our study may have reduced the statistical power of the analysis, limiting the ability to detect clinical differences between groups.

The primary limitation of this study was the relatively small number of patients who developed postoperative stiffness (n = 24), which may have resulted in a type II error. Consequently, several variables that showed a trend toward association with stiffness, such as female sex, age under 50, and partial-thickness tears, may have yielded false-negative results. A more extensive, multicenter case-control study with more cases and a rigorous methodological design could provide a more comprehensive understanding of the risk factors associated with EPS.

Additionally, the retrospective nature of the study design introduces a potential recall and selection bias, particularly in the collection of clinical variables, which relied exclusively on medical records and patient reports. The inclusion of patients from a single tertiary center limits the generalizability of the findings, as population characteristics and institutional practices may not reflect other settings. Another significant limitation was the absence of detailed laboratory assessments for conditions such as diabetes mellitus and hypothyroidism, which may have led to the inclusion of undiagnosed or poorly controlled cases. Finally, the lack of control over variables related to the rehabilitation process, such as adherence to the postoperative protocol and the quality of exercises performed, restricted the ability to assess the impact of these factors on outcomes. These aspects highlighted the need for prospective, multicenter studies with greater methodological control to understand the causes of postoperative stiffness further. Given the limited number of events and the resulting imprecision, the absence of statistically significant associations should not be interpreted as evidence of no effect; clinically relevant differences cannot be excluded, and the findings should be interpreted with caution.

This study had several strengths. Our sample is more significant than most similar studies on postoperative stiffness, which strengthened the reliability of our findings and reduces the influence of sampling bias. Standardizing the surgical and rehabilitation protocols also minimized biases related to technical differences or postoperative management, ensured greater homogeneity in outcome evaluation. Finally, the clear and objective definition of postoperative stiffness, based on well-established criteria applied by a single experienced evaluator, enhanced the accuracy of outcome identification and reduced inconsistencies in data collection.

This study did not identify statistically significant associations between clinical factors (sex, age, diabetes, hypertension, dyslipidemia, or hypothyroidism) or structural factors (tear size, retraction, or fatty degeneration) and the development of EPS after ARCR. The incidence of stiffness was 6.3%, consistent with rates reported in the literature.

## Conclusions

These findings reinforce the multifactorial nature of postoperative stiffness and the current lack of consistent predictive markers. From a clinical perspective, the absence of strong associations highlights the importance of individualized rehabilitation protocols and close postoperative monitoring rather than reliance on baseline risk factors. Future multicenter prospective studies with larger sample sizes and standardized criteria are needed to clarify potential associations and guide preventive strategies for this complication.
